# Radiotherapy in patients with brain metastases with and without concomitant immunotherapy: comparison of patient outcome and neurotoxicity

**DOI:** 10.1007/s12672-024-01560-6

**Published:** 2024-11-15

**Authors:** Natalie Elyan, Philipp Schwenkenbecher, Lea Grote-Levi, Jan-Niklas Becker, Roland Merten, Hans Christiansen, Thomas Skripuletz, Diana Steinmann, Nora Möhn

**Affiliations:** 1 Department of Radiotherapy, Hannover Medical School, Hannover, Germany; 2 Department of Neurology, Hannover Medical School, Carl-Neuberg-Str. 1, 30625 Hannover, Germany

**Keywords:** Immune checkpoint inhibitors, Radiotherapy, Neurotoxicity, Steroids

## Abstract

**Background/Aim:**

Recently, immune checkpoint inhibitors (ICI) have been added to the treatment of brain metastases. While combining radiotherapy and ICI can enhance therapeutic effects, it might also increase the risk of severe autoimmune adverse events. This retrospective study aims to compare treatment responses and neurotoxicity in patients treated with radiotherapy alone versus those receiving a combination of radiotherapy and ICI.

**Patients and methods:**

All patients with brain metastases who received radiotherapy at Hannover Medical School from 2017 to 2019 were included. The medical reports of all study participants were evaluated. Patients who received radiotherapy alone and those who received a combination of radiation and ICI were compared.

**Results:**

A total of 248 patients were analyzed, with the most common tumor types being non-small cell lung cancer (NSCLC) and malignant melanoma. Half of the patients received whole-brain radiotherapy (WBRT) and the other half stereotactic radiotherapy (SRT). Of these, 29 patients received concurrent immunotherapy and radiotherapy, 30 completed immunotherapy before radiotherapy, and 29 started ICI after completing radiotherapy. Two cases lacked information on the duration of immunotherapy. Overall survival post-initial tumor diagnosis within the total cohort was 52 months, with significantly worse survival for patients with multiple brain metastases (p = 0.020). No significant differences in survival or incidence of neurological adverse events were observed between patients with or without ICI.

**Conclusion:**

Combining radiotherapy and ICI did not significantly increase neurotoxicity or improve survival in this cohort, though the heterogeneity of the subgroups limits the generalizability of these findings.

## Introduction

Metastatic tumor disease is a severe diagnosis, particularly when it involves cerebral metastases, which significantly reduce overall survival by presenting unique challenges to the immune system's antitumor response [1–3]. In advanced malignant melanoma, brain metastases occur in approximately 50% of patients, leaving them with a life expectancy of only a few months [1, 2]. Similarly, in other cancers such as non-small cell lung cancer (NSCLC), renal cell carcinoma, and head and neck cancers, the prognosis remains poor once brain metastases are diagnosed [4]. Radiotherapy (RT), whether alone or post-surgery, remains a cornerstone of treatment for cerebral metastases. While whole-brain radiotherapy (WBRT) was once the standard, stereotactic radiotherapy (SRT) is now more commonly used due to its lower rates of neurocognitive impairment, even in cases of multiple metastases [5–7].

The advent of immune checkpoint inhibitors (ICI) has marked a significant advancement in the treatment of metastatic cancers [8]. Since the approval of antibodies targeting PD-1, PD-L1, and CTLA-4 for various metastatic tumors [9–11], ICI have also been incorporated into the treatment of brain metastases, often in combination with RT. While systemic therapies frequently fail to penetrate the blood–brain barrier (BBB), ICI have demonstrated partial responses in cerebral metastases [12]. Moreover, preclinical studies suggest that RT can even enhance the immune response by releasing tumor-associated antigens, thereby activating CD8 + cytotoxic T cells, which can cross the BBB and potentially overcome the brain's immune compartmentalization [13–15].

Despite these promising effects, the potential for adverse events from both treatments must be acknowledged. In addition to the known risks of late cognitive impairment and focal radionecrosis, RT can also cause acute or subacute neurotoxicities [16]. For instance, acute encephalopathy may occur shortly after RT, presenting with symptoms such as headache, nausea, vomiting, and impaired consciousness, likely due to BBB disruption [17–19]. Subacute effects, thought to be linked to transient demyelination, can manifest as somnolence syndrome, cognitive impairment, or subacute rhombencephalitis within weeks to months after RT [20]. Late effects like radionecrosis may arise even years after RT.

While ICI have proven highly effective, they also carry the risk of autoimmune side effects, including rare but serious complications affecting the central nervous system (CNS) [21]. Recent research has identified three clinical phenotypes of ICI-associated CNS syndromes: limbic encephalitis, meningoencephalitis, and cerebellitis [22–24]. Although CNS-related symptoms are rare compared to adverse events in other organ systems, the combination of RT and ICI could hypothetically increase the risk of neurotoxicity. However, a recent meta-analysis found no significant increase in grade 3–4 autoimmune toxicity when combining RT and ICI, compared to ICI alone, although the study did not specifically address neurotoxicity or factors like dexamethasone dosage [25]. Additionally, in a pooled analysis of 68 studies from the US Food and Drug Administration (FDA) database, the administration of ICI within 90 days of RT did not appear to be associated with an increased risk of serious adverse events. The authors postulated that the administration of ICI within 90 days of RT appears to be safe [26].

Data on neurotoxicity associated with combined RT and ICI remain limited. In this single-center, retrospective study of 248 patients, we examine both treatment responses and neurotoxicity in those treated with RT alone versus those receiving a combination of RT and ICI. These findings aim to expand the evidence base and support future studies on this relevant clinical topic.

## Patients and methods

Patients with brain metastases who had undergone radiation therapy at the Department of Radiotherapy at Hannover Medical School between 01/2017 and 12/2019 were included in the analysis. The patients’ electronic medical records were used to extract the data. All individuals who received radiation therapy with or without an additional treatment with immune checkpoint inhibitors before, during or after radiotherapy of brain metastases were enrolled. Here, the group with combination of ICI and radiation included all individuals treated with ICI after initial diagnosis of brain metastases up to and including 12/2019. For each patient treated the following items were examined: age at the time of radiotherapy, gender, primary tumor, histology and localization of primary tumor, previous oncological therapies, time between diagnosis of primary tumor and the appearance of brain metastases, number of brain metastases, date of surgical treatment of brain metastases, neurological adverse events that occurred during radiotherapy, start and end date of therapy with immune checkpoint inhibitors (ICI), name of ICI product, date of death or date of last follow-up visit, systemic progress/systemic therapy response, therapeutic response with respect to brain metastases (assessed via brain MRI). Cerebral tumor progression was defined as recurrence of tumor in radiation field or outfield at any other site of the brain. The follow up MRI were evaluated with the assistance of an in house radiologist.

Neurologic adverse events were retrospectively classified into grades of severity 1–5 according to the common terminology criteria of adverse events (CTCAE) (Table 1). In addition, the dose of dexamethasone applied was determined in each patient. The different radiation modalities included whole brain radiation (WBRT) and hypofractionated stereotactic radiation therapy (SRT). In case of stereotactic radiation therapy different fractionation schemes have been applied, except of single fraction radiosurgery. A group comparison was made between patients with and without additional immunotherapy. The group of patients treated with radiotherapy and immunotherapy was further divided into the subgroups with simultaneous immunotherapy or immunotherapy before or after radiotherapy.

**Table 1:** Definition of common terminology criteria of adverse events (CTCAE) grades

CTCAE grades	Definition
Grade 1	Mild; asymptomatic or mild symptoms; clinical or diagnostic observations only; intervention not indicated
Grade 2	Moderate; minimal, local or noninvasive intervention indicated; limiting age-appropriate instrumental ADL
Grade 3	Severe or medically significant but not immediately life-threatening; hospitalization or prolongation of hospitalization indicated; disabling; limiting selfcare ADL
Grade 4	Life-threatening consequences; urgent intervention indicated
Grade 5	Death related to AE

*ADL* activities of daily living, *AE* adverse events

The study was approved by the local Ethics Committee at Hannover Medical School (Ethics Approval Number: 10950_BO_K_2023). All patients provided written informed consent for the use of their data for scientific purposes.

### Statistical analysis

Statistical analyses and graphical illustrations were prepared using spss (version 27) and graphpad prism (version 10). For group comparisons in terms of overall survival, Mann–Whitney-U test was used. Binary logistic regression for metric and nominal variables (categorical predictor) was applied to analyze the influence of different factors on the dependent variable death/non-death as well as local control. The correlation between severity of neurological symptoms and other factors such as age, sex, or radiation dose was calculated using the chi-square test. Kaplan–Meier curves were used to estimate overall survival (OS).

## Results

### Patients’ characteristics

In the specified period, a total of 248 patients with brain metastases underwent brain irradiation at our center. Retrospective data could be analyzed from all of them and details on patients’ characteristics are summarized in Table 2. Median age of the total cohort was 62 years (range 19–87 years) with a male/female ratio of 1:1. The most common underlying tumor entity was non-small cell lung cancer (NSCLC) (n = 69, 28%), followed by malignant melanoma (n = 53, 21%). Brain metastases were detected within 2.9 years (range: 0–26 years) after diagnosis of the primary tumor. The vast majority of patients within the cohort received local or systemic therapies when being diagnosed with the underlying tumor disease. Locoregional treatment of the detected brain metastasis included surgery at one or more metastatic sites (n = 89, 36%) followed by radiation therapy of the tumor bed. One hundred and fifty-nine patients (64%) received primary radiation therapy of the CNS lesions. Thirty-six percent of patients (n = 90) were treated with ICI in addition to radiotherapy. In 29 (32.2%) cases, patients received immunotherapy and radiation of brain metastasis simultaneously, whereas 30 patients (33.3%) already finished ICI prior to radiotherapy and 29 patients (32.2%) received ICI after completion of radiation. Patients with ICI before RT received immunotherapy a median of 305 days before radiation, while the period from ICI after RT was a median of 93 days. In two cases (2.2%), detailed information on the start of ICI therapy was missing. The anti-PD-1 antibodies pembrolizumab and nivolumab were used most frequently (n = 52; 57.8%). In 19 cases (21.1%), patients were treated with a combination therapy of anti-PD-1 and anti-CTLA-4 antibodies. Both groups -with combined therapy and with radiotherapy alone- differed significantly with respect to gender distribution and underlying tumor entities, among other factors. In the ICI group, there were significantly more male patients (p = 0.001) and more patients with NSCLC (p = 0.019). In addition, patients in the group with complementary immunotherapy received significantly higher single doses during radiotherapy (Table 2).

**Table 2:** Patient characteristics and treatment parameters

Patient characteristic and treatment parameters	All patients (n = 248)	Radiotherapy and ICI (n = 90)	Radiotherapy only (n = 158)	P-value
Age at diagnosis of brain metastasis (years), median (range)	62 (19–87)	62 (28–82)	62 (19–87)	0.398
Sex, n (%)				
Male	123 (49.6)	58 (64.4)	65 (41.1)	**0.001**
Female	125 (50.4)	32 (35.6)	93 (58.9)	
Primary tumor entity, n (%)				
Non-small cell lung cancer	69 (27.8)	33 (36.7)	36 (22.8)	**0.019**
Small cell lung cancer	23 (9.3)	1 (1.1)	22 (8.9)	**0.001**
Malignant melanoma	53 (21.4)	43 (47.8)	10 (6.3)	**0.001**
Renal cell carcinoma	8 (3.2)	6 (6.7)	2 (1.3)	**0.021**
Colorectal cancer and cancer of upper gastrointestinal tract	10 (4.0)	2 (2.2)	8 (5.0)	0.274
Breast cancer	44 (17.7)	2 (2.2)	42 (26.6)	**0.001**
Prostate cancer	4 (1.6)	0 (0)	4 (2.5)	0.128
Other cancer	37 (14.9)	3 (3.3)	34 (21.5)	**0.001**
Systemic and cerebral metastasis at time of tumor diagnosis, n (%)				
Primary cerebral + extracerebral	121 (48.8)	46 (51.1)	75 (47.5)	0.581
Secondary	120 (48.4)	42 (46.7)	78 (49.4)	0.682
Primary only cerebral	7 (2.8)	2 (2.2)	5 (3.2)	0.667
Treatment mode of primary tumor, n (%)				
Surgery	23 (9.3)	9 (10.0)	14 (8.9)	0.766
Surgery + chemotherapy	25 (10.1)	5 (5.6)	20 (12.7)	0.074
Chemotherapy alone (± radiation)	59 (23.8)	12 (13.3)	47 (29.7)	**0.004**
Primary chemotherapy + other systematic substances	30 (12.1)	19 (21.1)	11 (7.0)	**0.001**
Surgery + chemotherapy + other systematic substances	32 (12.9)	7 (7.8)	25 (15.8)	0.069
Surgery + other treatment modalities	30 (12.1)	17 (18.9)	13 (8.2)	**0.013**
Other treatment modalities	39 (15.7)	20 (22.2)	19 (12.0)	**0.034**
No treatment of primary tumor	10 (4.0)	1 (1.1)	9 (5.7)	0.078
Average time till diagnosis of brain metastasis, years (range)	2.92 (0–26)	2.50 (0–18)	3.15 (0–26)	0.285
Number of brain metastases at first diagnosis, n (%)				
Single metastasis	108 (43.5)	41 (45.6)	67 (42.4)	0.630
2–3 metastases	47 (19.0)	22 (24.4)	25 (15.8)	0.096
> 3 metastases ± meningeosis carcinomatosa	93 (37.5)	27 (30.0)	66 (41.8)	0.066
Patients with cerebral progress	71 (28.9)	38 (42.2)	33 (21.2)	**0.001**
Operative treatment of brain metastases, n (%)				
Surgery	89 (35.9)	25 (27.8)	64 (40.5)	**0.044**
No surgery	159 (64.1)	65 (72.2)	94 (59.5)	**0.044**
Treatment modality of first radiation, n (%)				
Stereotactic radiotherapy (SRT)	124 (50.0)	51 (56.7)	73 (46.2)	0.113
Whole brain radiation (WBRT)	124 (50.0)	39 (43.3)	85 (53.8)	0.113
Treatment modality of second radiation, n (%)				
Patients, n (%)	46 (18.5)	26 (28.9)	20 (12.7)	
SRT	31 (67.4)	18 (69.2)	13 (65.0)	0.598
WBRT	15 (32.6)	8 (30.8)	7 (35.0)	0.851
Treatment modality of third radiation, n (%)				
Patients, n (%)	11 (4.4)	4 (4.4)	7 (4.4)	
SRT	8 (72.7)	3 (75.5)	5 (71.4)	0.898
WBRT	3 (27.4)	1 (25.0)	2 (28.6)	0.898
Radiation dosage				
All modalities: single dose per fraction (Gy), mean (range)	4.0 (2.0–8.0)	4.5 (2.5–8.0)	3.5 (2–8)	**0.039**
All modalities: total dose (Gy), mean ± STD	31.7 ± 7.5	31.6 ± 7.2	35 ± 7.7	0.340
All modlities: EQD2 (alpha/beta: 10) mean ± STD	39.6 ± 8.6	34.25 ± 7.4	39.59 ± 9.1	0.818
All modalities: BED 10 mean ± STD	47.5 ± 10.3	41.1 ± 8.9	47.5 ± 10.8	0.667
SRT: Single dose per fraction (Gy), mean (range)	4.0 (2.5–8)	6 (2.5–8)	4 (2.5–8)	**0.034**
SRT: Total dose per fraction (Gy) ± STD	40 ± 7.2	32 ± 7.2	40 ± 7.7	**0.006**
SRT: EQD2 (alpha/beta: 10) mean ± STD	46.67 ± 7.2	41,34 ± 6.3	46.54 ± 7	**0.008**
SRT: BED 10 mean ± STD	56 ± 8.6	49.6 ± 7.6	56 ± 8.4	**0.009**
WBRT: Single dose per fraction (Gy), mean (range)	3.0 (2–8)	3 (2.5–8)	3 (2–4)	0.770
WBRT: Total dose per fraction (Gy) ± STD	30 ± 7.4	33,5 ± 7.1	30 ± 6.3	0.137
WBRT: EQD2 (alpha/beta: 10) mean ± STD	32.5 ± 8	36.29 ± 7.9	32.5 ± 6.8	0.071
WBRT: BED 10 mean ± STD	39 ± 9.7	43.55 ± 9.3	39 ± 8.1	**0.036**
Boost, n (%)				
Yes	64 (25.8)	22 (24.4)	42 (26.6)	0.711
No	184 (74.2)	68 (75.6)	116 (73.4)	0.711

Other systematic substances used: molecular targeted therapy, anti-angiogenic agents. Other treatment modalities: watch and wait strategy, tumor ablation procedures

*BED* biologically effective dose, *EQD2* equivalent dose of 2 Gy, *PRT* partial brain radiation therapy, *RT* radiotherapy, *SRT* stereotactic radiotherapy, *WBRT* whole brain radiation therapy

Bold letters indicate statistical significance

### Adverse events

In a total of 36 patients (40%) systemic autoimmune adverse events of ICI therapy were documented within the follow-up period. These included for example autoimmune colitis, hepatitis, pneumonitis, dermatitis, and hypophysitis. Ten (11%) patients suffered a progress either of their extracerebral tumor lesions or their CNS manifestation. In two cases (2%) the patients both experienced side effects of ICI treatment and a progress of their tumor disease, while 42 patients (47%) did not show any specific side effects.

In the group of patients who received concurrent or sequential radiotherapy and ICI treatment, 50 (56%) showed no neurological adverse events, while 11 patients (12%) experienced mild neurological deficits of CTCAE grade 1 (Fig. 2). Moderate neurological symptoms could be observed in 15 patients (17%) and in nine cases (10%) grade 3 neurotoxicity was documented (Fig. 2). In three patients (3%) no information about neurological adverse events could be obtained. Detailed information regarding neurological side effects in the different treatment groups can be obtained from Table 3.

**Table 3:** Frequency of neurological adverse events in the different treatment groups

Neurotoxicity n (%)	RT without ICI (n = 158)	Concomintant RT and ICI (n = 29)	ICI prior to RT (n = 30)	ICI after RT (n = 29)	Others* (N = 2)
Grade 0	84 (53.2)	16 (55.2)	17 (56.7)	16 (55.2)	1 (50.0)
Grade 1	16 (10.1)	0 (0)	6 (20)	5 (17.2)	0 (0)
Grade 2	34 (21.5)	9 (31.0)	2 (6.7)	4 (13.8)	0 (0)
Grade 3	22 (13.9)	4 (13.8)	2 (6.7)	3 (10.3)	1 (50.0)
Grade 4	0 (0)	0 (0)	1 (3.3)	0 (0)	0 (0)
No information on Neurotox	2 (1.3)	0 (0)	2 (6.7)	1 (3.5)	0 (0)

*ICI* immune checkpoint inhibitor, *RT* radiotherapy

^*^No information about beginning and ending of ICI therapy

The majority of patients without ICI treatment (n = 84, 53%) showed no neurological deficits during cerebral radiation therapy. In 16 (10%) and 34 (21%) patients mild (CTCAE grade 1) and moderate (CTCAE grade 2) neurological adverse events could be detected, respectively. Grade 3 neurotoxicity occurred in as many as 22 patients (14%) (Table 3, Fig. 2). Interestingly, corticosteroids were not used in 44% (n = 69) of patients in the group without ICI treatment. Only the administration of dexamethasone during radiotherapy was taken into account. Whether dexamethasone was started before radiotherapy was not assessed for the individual patients. The same applies to the cumulative dexamethasone dose within the sub-groups. Further information on the application of steroids in the cohort can be extracted from Table 4.

**Table 4:** Application of dexamethasone in the different treatment groups

Dexamethasone application, n(%)	RT without ICI (n = 158)	Concomitant RT and ICI (n = 29)	ICI prior to RT (n = 30)	ICI after RT (n = 29)	Others* (n = 2)
None	69 (43.7)	16 (34.0)	12 (40.0)	12 (41.4)	0
1–3 mg	5 (3.2)	0	0 (0)	1 (3.5)	0
4–5 mg	12 (7.6)	5 (10.6)	3 (10.0)	1 (3.5)	1 (50.0)
6–8 mg	16 (10.0)	8 (17.0)	6 (20.0)	5 (17.2)	0
> 8 mg^#^	48 (30.4)	13 (27.7)	6 (20.0)	7 (24.1)	1 (50.0)
No information on dosage	8 (5.1)	5 (10.6)	3 (10.0)	3 (10.3)	0

*ICI* Immune checkpoint inhibitor, *RT* radiotherapy

^*^No data concerning the beginning and end of ICI therapy

^#^dexamethasone daily dose of more than 8 mg up to a maximum of 40 mg per day

### Radionecrosis rate

In the follow-up MRI, radionecrosis was detected in two patients (1%) within the group of patients who were treated with radiation alone (n = 158). Both received hypofractionated stereotactic radiation and one of these patients had a reradiation of the same anatomical region with a high single dose of 8 Gy per fraction. None of the patients with ICI therapy exhibited radionecrosis.

### Tumor outcome

The median follow-up of the overall cohort was 4 months after start radiotherapy (range: 0–47 months). Within the group receiving both radiation and ICI treatment, 41 patients (46%) suffered a systematic progress and 38 patients (42%) showed progress of brain metastases either within or outside the radiation field. Detailed information on local control can be obtained from Table 5. In 29 cases (32%) the tumor disease remained stable, six patients (7%) experienced a remission of their systemic tumor spread and five patients (6%) showed a mixed response. In 30 persons (33%) who had been treated with a combination of ICI and radiotherapy, a steady-state or remission of the cerebral metastases was observed.

**Table 5:** Assesment of local control depending on the treatment mode

Cerebral progression	RT without ICI (n = 158)	Concomitant RT and ICI (n = 29)	ICI prior RT (n = 30)	ICI after RT (n = 29)	Others* (n = 2)
Infield	9 (5.7)	3 (10.3)	7 (23.3)	3 (10.3)	0 (0)
Outfield	13 (8.2)	3 (10.3)	1 (3.3)	3 (10.3)	0 (0)
Infield + outfield	14 (8.9)	5 (17.2)	5 (16.7)	7 (24.1)	1 (50.0)
Steady state or remission	37 (23.4)	14 (48.3)	5 (16.7)	10 (34.5)	1 (50.0)
Pseudoprogression	0 (0)	1 (3.4)	0 (0)	0 (0)	0 (0)
Radionecrosis	2 (1.3)	0 (0)	0 (0)	0 (0)	0 (0)
Abscopal effect	0 (0)	1 (3.5)^#^	0 (0)	0 (0)	0 (0)
No follow up MRI	85 (53.8)	3 (10.3)	12 (40.0)	6 (20.7)	0 (0)

*ICI* Immune checkpoint inhibitor, *RT* radiotherapy

^*^No data concerning the beginning and end of ICI therapy

^#^Patient showed both remission and abscopal effect

In the group of patients with radiation alone, 36 patients (23%) had an in-field or out-field progress of their brain metastases, while 36 of them (22%) showed a steady-state or remission regarding CNS-tumor manifestation (Table 5). However, in 85 patients (54%) no further information on outcome of cerebral metastases could be obtained.

**Fig. 1:**
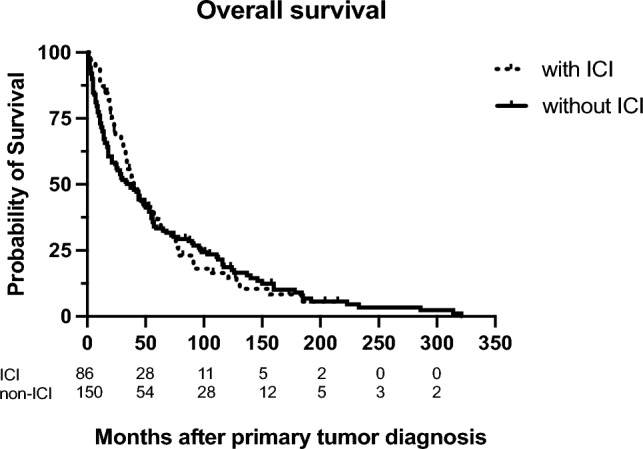
Overall survival of patients treated with a combination of radiotherapy and immune checkpoint inhibitors (dotted curve) and patients treated with radiotherapy alone, p = 0.14

Seventy-two patients (80%) of the ICI and radiation combination therapy group died, while 14 (16%) where still alive at the time of data analysis. The average overall survival of patients in this cohort who died within the observation period was 46.5 months after primary tumor diagnosis. In four cases, no current information on survival status could be obtained. Regarding the patient group that did not receive ICI treatment, 135 persons (85%) died showing a mean overall survival of 54.6 months (range: 1–321) and 15 (9.5%) survived to the time of data collection, while the outcome was unknown in eight cases. Figure 1 illustrates that overall survival did not significantly differ between the two treatment groups (p = 0.14).

### Dependence of outcome on patient-, tumor-, and treatment-characteristics

In a next step, various factors were investigated with regard to their influence on patient survival. Using binary logistic regression, it could be determined that melanoma (OR = 0.380) and renal cell carcinoma as underlying tumor disease (OR = 0.215) were associated with a lower risk of death. In contrast, neither the age at the start of radiotherapy, nor the total radiation dose administered had a significant influence on survival (Table 6). Moreover, cohort survival was not significantly related to whether or not immunotherapy was used in addition to radiation. Patients with multiple cerebral metastases showed a significantly elevated risk for the outcome death (OR = 3.278).

**Table 6:** Univariate results of logistic regression on mortality endpoint

Variable	p-value	Odds ratio	95% confidence interval	
			Lower value	Upper value
Sex (male)	0.106	1.956	0.868	4.409
Age	0.496	0.988	0.955	1.022
Underlying tumor (melanoma)	**0.022**	0.380	0.166	0.869
Underlying tumor (renal cell carcinoma)	**0.043**	0.215	0.048	0.950
Immunotherapy applied	0.161	1.750	0.800	3.827
Multiple cerebral metastases	**0.020**	3.278	1.203	8.934
Total radiation dose	0.260	1.033	0.976	1.094
High dose dexamethasone > 8 mg	0.244	1.824	0.664	5.011

Bold letters indicate statistical significance with p < 0.05

With regard to potential determinants of the local control of irradiated brain metastases, it was observed that only the fact whether a boost was applied or not had a significant influence. Patients who received a boost had a significantly increased chance (OR = 2.857) of not suffering progression of brain metastases (Table 7). In comparison, the radiotherapy modality, the total radiation dose or whether additional immunotherapy was used had no impact on local control. However, it must be mentioned that no follow-up cMRI was available in 106 cases.

**Table 7:** Univariate results of logistic regression on endpoint local control

Variable	p-value	Odds ratio	95% confidence interval	
			Lower value	Upper value
Immunotherapy applied	0.340	1.379	0.713	2.670
Concurrent immunotherapy	0.462	1.444	0.542	3.851
Multiple cerebral metastases	0.389	0.740	0.372	1.470
Total radiation dose	0.364	0.978	0.931	1.027
EQD2 (alpha/beta: 10)	0.306	0.978	0.936	1.021
BED (alpha/beta: 10)	0.218	0.978	0.943	1.014
Radiation mode -WBRT	0.501	1.255	0.648	2.434
Boost applied	**0.010**	2.857	1.288	6.337

Bold letters indicate statistical significance with p < 0.05

*BED* biologically effective dose, *EQD2* equivalent dose of 2 Gy, *WBRT* whole brain radiation therapy

### Factors influencing the occurrence of neurological adverse events

**Fig. 2:**
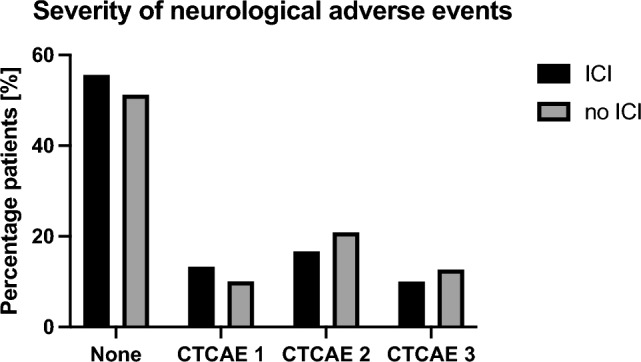
Illustration of the respective severity of neurotoxicity depending on the therapeutic regimen. CTCAE: common terminology criteria of adverse events

In addition, potential factors influencing the occurrence or severity of neurological complications in patients with cerebral metastases were investigated. It could be determined that neither the age of the patients (p = 0.735), their sex (p = 0.134), nor the underlying tumor disease (p = 0.069) or the radiation modality (p = 0.669) correlated with the severity of neurological complications. ICI therapy caused no statistically significant difference in the severity of neurotoxicity (p = 0.724) (Fig. 2).

The number of cerebral metastases at initial diagnosis of cerebral metastatic tumor disease correlated significantly with the severity of neurological symptoms (p = 0.016) and the dose of dexamethasone used increased depending on the severity of neurological symptoms (p < 0.001).

## Discussion

In this series of 248 patients with cerebral metastases who received radiotherapy, we compared those with additional ICI therapy and patients with radiation alone regarding tumor outcome and neurotoxicity. As this is a retrospective study, the two groups differed significantly with regard to distinct baseline characteristics, for example, in terms of gender distribution or the frequency of underlying tumor diseases (Table 2). No significant difference regarding the overall survival of patients with combination therapy and those with radiation alone could be detected (Fig. 1). We demonstrated that one of the factors influencing overall survival in this cohort was the underlying tumor entity (Table 6). Patients with melanoma (OR = 0.380) and renal cell carcinoma (OR = 0.215) showed an decreased risk of death. Due to the heterogeneity of the two patient groups with and without ICI therapy with regard to the distribution of the underlying tumour entities, the potential effect of the additional immunotherapy on overall survival can presumably only be assessed to a limited extent. However, the study also shows that immunotherapy had no positive effect on local control in this cohort. In contrast, the application of a boost during radiotherapy increased the chance of local control of the brain metastases (OR = 2.857). Similar results were previously obtained in a phase III randomised trial (RTOG 9508), which demonstrated that patients with brain metastases who received whole brain radiation therapy (WBRT) followed by a stereotactic radiosurgery boost had better complete response rates and improved local control compared to those who received only WBRT [27].

In future studies of combination therapy of ICI and radiotherapy, it is essential to consider the timing of immunotherapy and the fractionation regimens of radiation dose [28]. It has already been shown that it might be relevant whether the systemic therapy is applied concurrent to the cerebral irradiation or time-delayed. Particularly regarding the fact that radiation therapy induces immunological changes that may improve the therapeutic effect of ICI [29]. In our cohort no significant difference was observed regarding the timing of immunotherapy (Table 7). However, it should be noted that the time between ICI and RT in the cohort studied was quite long in some cases (median time from ICI to RT: 305 days; median time from RT to ICI: 93 days). Nevertheless, irAE after ICI lasting up to 1 year have been described. In addition, a large pooled analysis of studies on the influence of ICI on the risk of adverse events after RT also considered a period of 90 days [26]. Additionally, the response rate of brain metastasis and distant tumor control in case of combination therapy may differ depending on the antibody applied [30].

Besides its therapeutic potential, the combined therapy of radiation and ICI for cerebral metastases may carry the risk of increased neurotoxicity [31], especially for those patients who have already received prior cerebral radiotherapy during their oncological history [31]. Besides the potential autoimmune side effects of ICI therapy, the risk of radionecrosis must be considered. In principle, long-term side effects after brain irradiation are playing an increasingly important role, as improved treatment methods are prolonging patient survival. These side effects, which are detected in post-treatment imaging, include radiation necrosis and blood–brain barrier disruptions, which themselves can cause neurological symptoms [32, 33]. Current publications discuss whether the blood–brain barrier disruption caused by radiotherapy increases the risk of ICI-induced autoimmune encephalitis and whether, in turn, the effects of ICI therapy enhance the risk of radiation necrosis [25]. This question has not yet been clearly addressed and larger prospective studies on this topic are lacking. In addition, the diagnosis of radiation necrosis itself and the differentiation from tumour necrosis or tumour progression is extremely challenging and standardised guidelines in this regard do not yet exist [32].

In the present study, radionecrosis was detected in only two patients who received radiotherapy without ICI. In these cases, both patients were treated with a hypfractionated stereotactic radiotherapy and one of them even had a reirradiation with a high single dose per fraction. Thus, there is no indication in this cohort that additional ICI therapy leads to an increased rate of radiation necrosis, although it must be mentioned that no follow-up MRI was available in 21 patients with combination therapy of ICI and radiotherapy. Commonly, neurotoxicity during brain irradiation is not assessed in a standardized manner [34]. Therefore it can only be speculated whether the combination of ICI and radiation leads to more neurological adverse events. When comparing both subgroups in this retrospective analysis, we did not detect any difference in the occurrence of neurological adverse events in general (p = 0.724) and also related to the respective severity levels (Fig. 2). These results are in line with a recent large pooled analysis of 68 studies from the FDA database that examined the influence of ICI on the adverse events rate after brain radiation [26]. Additionally, no difference was found regardless of whether the patients received ICI therapy concomitantly with radiotherapy or sequentially (Table 5). A total of 39% of patients receiving combination therapy and 45% of patients receiving radiotherapy alone experienced neurological symptoms of varying severity. In the literature, the incidence of acute neurotoxicity under brain radiation is reported to be up to 50% [17]. Neurological CNS adverse events associated with ICI therapy are overall very rare [35], so additional immunotherapy may not have a numeric effect on the incidence of neurological symptoms in the studied cohort. In addition, whether WBRT or another modality was used, did not affect the frequency of neurotoxicity in this cohort (p = 0.669). Only the number of cerebral metastases had a significant effect on the incidence of higher-grade neurological adverse events (p = 0.016). However, this may be related to the fact that neurological symptoms were caused by the metastases themselves and not by the therapy. In future studies, it is essential to differentiate whether a neurological symptom is tumor-related or therapy-related. The recording of the dexamethasone dosage also provides only limited help in this regard, since steroids are used not only for autoimmune neurological adverse events but also for anti-edematous therapy to alleviate neurologic symptoms caused by the metastases themselves [36]. This is supported by the fact that steroid dose correlated with the number of metastases (p = 0.001). Basically, it should be noted that steroid use in patients with combination therapy did not differ significantly from those with radiation alone (p = 0.063). However, it needs to be pointed out that in this study only the daily steroid dose during radiotherapy was recorded and not any intake prior to it. It is also not possible to draw any conclusions about the cumulative steroid dosage of the subgroups. Overall, though, we can conclude that, subject to the aforementioned limitations, patients receiving combination therapy in our cohort did not show an increased risk of relevant neurotoxicity during treatment. This is in line with other previous studies [26, 37, 38].

When initiating therapy for brain metastases, whether radiotherapy alone or combined with ICI therapy, patients should be well-monitored neurologically. The present evaluation shows that especially patients with multiple cerebral metastases require special (neurological) attention. Since these patients also have an increased need for steroids which could also negatively affect the efficacy of immunotherapeutic agents such as ICI, alternative anti-edematous therapies, such as bevacizumab, should be considered for long term toxicities after radiotherapy [39].

Despite the relatively large number of individuals included, this study has some limitations:

Due to the retrospective nature of the data, certain aspects such as the type (acute toxicity vs. late toxicity) and severity of neurotoxicity can only be assessed to a limited extent, as the evaluation is dependent on the quality of the previous documentation. The same applies to the determination of overall survival and local control. In principle, no causal conclusions can be drawn from a retrospective study. Additionally, it should be noted that the groups compared are very heterogeneous, both in terms of the number of patients and their characteristics. This also influences the results and their interpretation. In particular, the influence of the different tumor entities on survival has to be carefully considered in this context. Furthermore, the differences in systemic control between patients treated with RT alone versus RT plus ICI (Table 2) could introduce a selection bias and the imbalance in single doses of radiation and surgical interventions between the two groups is a potential confounding factor as these differences might affect the comparison of outcomes between the RT alone and RT + ICI group. Future research could adopt a case–control methodology to strengthen the findings and provide a clearer answer to the scientific questions. In any case, future studies will need a much more homogeneous patient population in order to measure the impact of ICI on survival and statistical adjustments to account for potential confounding factors.

However, this work is an important step toward a better understanding of the processes involved in the treatment of brain metastases and should lay the groundwork for a future prospective study to assess neurotoxicity during cerebral irradiation with and without ICI therapy.

## Conclusion

In general, the combination of ICI treatment and radiation has shown to improve overall survival in patients with brain metastases, while it may also harbor the risk of severe autoimmune side effects. In our cohort, the combinatory therapy did not lead to an increase in neurotoxicity and additional ICI application did not improve patient survival or local control which may be due to the unequal subgroup distribution. It can be stated that recording neurological symptoms during radiotherapy is of utmost importance and a more detailed neurological assessment in the future may lead to earlier detection of potential neurological adverse events of immunotherapy, thus improving patients' quality of life and outcome.

## Data Availability

The data that support the findings of this study are not openly available due to reasons of sensitivity and are available from the corresponding author upon reasonable request. Data are located in controlled access data storage at Hannover Medical School.
